# Foot-at-risk among adult outpatients with diabetes mellitus in Bowen University Teaching Hospital, Ogbomoso, Nigeria

**DOI:** 10.11604/pamj.2023.46.106.39397

**Published:** 2023-12-14

**Authors:** Adeola Ebenezer Idowu, Isaac Olusayo Amole, Adewumi Ojeniyi Durodola, Stephen Adesope Adesina, Olatayo Araade Idowu, Adepeju Olatayo Adegoke, Philip Bamigboye, Olufemi Timothy Awotunde, Akintayo David Ola Olorun

**Affiliations:** 1 Department of Family Medicine, Bowen University Teaching Hospital, P.O. Box 15, Ogbomoso, Nigeria,; 2 Department of Family Medicine, Bowen University Iwo and Bowen University Teaching Hospital, P.O. Box 15, Ogbomoso, Nigeria

**Keywords:** Foot-at-risk, neuropathy, peripheral arterial disease, glycemic control

## Abstract

**Introduction:**

the rising prevalence of diabetes mellitus (DM) around the world has dramatically increased the number of people bearing the complications of this potentially incapacitating disease. One of these complications is foot ulcers that may result in amputation. This study sets out to determine the profiles of the “foot-at-risk” for ulceration and the associated socio-medical factors in DM patients.

**Methods:**

this study was conducted at Bowen University Teaching Hospital, Ogbomoso, Southwest, Nigeria. This was a descriptive cross-sectional study comprising 299 outpatient adults aged 18 years and above with diabetes mellitus of at least 6 months in duration. Comprehensive Foot Examination and Risk Assessment tool was used to identify the foot-at-risk categories of the participants. Data analysis was done using Statistical Package for Social Sciences (SPSS) version 20.

**Results:**

the prevalence of foot-at-risk among the participants was 64.9% (194). Among the 194 participants with foot-at-risk, 35.1% (105) belonged to the foot-at-risk categories 0, 37.8% (113) in category 1, 16.1% (48) in category 2, and 11.0% (33) in category 3. Other factors that had a statistically significant association with foot-at-risk included; age, religion, level of education, duration of diabetes, history of smoking, and glycemic control.

**Conclusion:**

foot-at-risk was found to have an alarming prevalence among the participants. In addition, the level of glycemic control in this group was unacceptably poor. Clinicians need to intensify preventive measures like foot screening and health education to prevent foot ulcerations, which may result in limb amputation in DM patients.

## Introduction

Diabetes mellitus (DM) is fast assuming a pandemic status with an alarming increase in its prevalence rate [1]. The rising prevalence of diabetes around the world has dramatically increased the number of people bearing its complications. One of such complications is the diabetic foot disease (DFD) [2]. It is defined as a foot affected by ulceration that is associated with neuropathy and/or peripheral arterial disease of the lower limb in a patient with diabetes. The burden of DFD is so intense that one person undergoes amputation every 30 seconds around the globe [3].

Mortality rates after diabetic foot ulceration and amputation are high, with up to 70% of people dying within 5 years of having an amputation and around 50% dying within 5 years of developing a diabetic foot ulcer [4]. Up to 85% of these amputations can be prevented with adequate measures [5]. It is therefore necessary that screening of patients with DM to identify foot-at-risk of ulceration and associated risk factors could be instituted to reduce the risk of foot ulceration and foot amputation [4]. Risk identification is fundamental for effective preventive management of the foot in people with DM [6]. This involves identifying foot-at-risk of ulceration as well as assessment of their glycaemic control. For the purpose of this study, “foot-at-risk” refers to the foot in DM patient with intact skin which may have bony or soft tissue deformities, features of neuropathy and/or vasculopathy, and a history of previous ulceration and/or amputation [6]. It is important to note that when appropriate actions are instituted after identification of foot-at-risk of ulceration, the rate of DFD will significantly reduce.

Unfortunately, according to our research, there has not been any recent report on the foot-at-risk in the Southwestern part of Nigeria. Hence, this study was conducted to assess the profile of foot-at-risk and associated socio-medical factors in diabetic patients accessing outpatient care in Bowen University Teaching Hospital, Ogbomoso, South-west Nigeria.

## Methods

**Setting:** the study was conducted in Bowen University Teaching Hospital (BUTH), Ogbomoso, South-west Nigeria. It was formerly called Baptist Medical Centre and was established in 1907 and upgraded to a teaching hospital in 2009. It has over 400 beds and the hospital sees over 50,000 outpatients and 10,000 in-patients per year.

**Study design:** this was a cross-sectional study conducted between March and June 2018. Systematic sampling technique was used for the selection of participants and 299 participants were recruited for the study.

**Study size determination:** the sample size for the study was calculated using the statistical formula [7].


$$n={\text{Z}}^{2}{\text{pq/d}}^{2}$$

Where: n = desired sample size, Z = Two standard deviations usually set at 1.96 which corresponds to 95% confidence level. p = the proportion in the target population estimated to have a particular characteristic. q = the proportion of the population not involved in the study i.e. 1-p. d = the degree of accuracy desired usually set at 0.05. Using the prevalence of diabetic foot-at-risk of 41.5% in South-west Nigeria [6]. Therefore, n = 373.1 approximated to 373.

However, the population of patients with diabetes mellitus that was seen in the last one year from the records department was 1200. Since the study population is < 10,000, the sample size was adjusted using the formula; [7].


nf=n/(1+(n-1)/N)

Where: nf = Desired sample size when population is less than 10,000. n = Desired sample size when population is greater than 10,000. N = Estimate of the population size = 1200. Therefore, nf = 373/ (1+ (373-1)/1200) ≈ 285. An allowance of 5% (14.3) was given for poorly completed questionnaire and missing test results. This was added to the desired sample size to give a total of 299.

**Study participants and eligibility criteria:** consenting patients aged 18 years and above with diabetes mellitus that have been on treatment for at least 6 months were recruited for the study. Pregnant patients, cognitively impaired patients and patients with active foot ulcers were excluded from the study.

**Variables:** for each of the recruited subjects, data collected were: socio-demographic and medical characteristics which included age, gender, marital status, religion, level of education, social class, ethnicity, residence, smoking history, duration of DM and comorbid medical illness; physical examination using comprehensive foot examination and risk assessment used to identify and categorize foot at risk; venous blood sample was taken for HbA1C measurement (glycaemic control) using A1CNow®+ system.

**Data sources/measurement:** the comprehensive foot examination and risk assessment was used for identification and categorization of foot-at-risk. It is a validated tool for categorizing foot-at-risk in patient with DM [8]. It included three (3) criteria which were; Foot inspection - for identifying foot deformities, Neurologic assessment - for identifying loss of protective sensation (LOPS) and Vascular assessment - for identifying peripheral arterial disease (PAD).

**Foot inspection:** the feet of participants were inspected under bright light for the presence of foot deformities and callous.

**Neurologic assessment:** a 10-g Semmes-Weinstein monofilament test and 128-Hz tuning fork test were used to identify LOPS as recommended by ADA [9]. At least one abnormal test suggested LOPS. The 10-g Semmes-Weinstein monofilament (manufactured by Atlas Biomechanics, Scottsdale, USA) was used to test for pressure perception. This was done in four sites (1st, 3rd, and 5th metatarsal heads and plantar surface of the distal hallux) each on both feet. With patient´s eyes opened, the device was placed perpendicular to the skin of the palm with pressure applied until the monofilament buckles (it was held in place for ~1 s and then released) after the sensation of pressure has been recognized by patient, the sensation in the foot was tested with patient´s eyes closed. Patient was expected to respond “yes” when the monofilament was applied to a particular site and also to identify the correct site of placement. The vibration test was done by using a 128-Hz tuning fork. Vibration perception was tested over the bony prominence situated at the dorsum of the foot corresponding to the head of the first metatarsal. With the patient lying supine and eyes closed, the stylus of the tuning fork was placed over the dorsal hallux. This process was initially demonstrated on the upper limb for the patient to appreciate the buzzing sensation.

**Vascular assessment:** the ankle-brachial index (ABI) measurement was performed by measuring the systolic blood pressure from both brachial arteries and from both posterior tibial arteries after the patient has been at rest in the supine position for 10 minutes. The systolic pressures were recorded with a hand-held 8-mHz Vascular Doppler (True Sonotrax Vascular Doppler manufactured by Edan Instrument Inc, China) and Accoson® mercury sphygmomanometer manufactured by Dekamet Accoson, England with appropriate cuff size. In measuring the brachial pressure, the patient was placed in supine position and blood pressure cuff placed around the arm. Ultrasound gel was applied to the antecubital fossa over the brachial pulsation. The transducer of the vascular Doppler was placed on the gel and its position was adjusted to the area with maximum signal intensity. The cuff of the sphygmomanometer was inflated to about 20mmHg above the expected systolic blood pressure of the patient. Then, the cuff was slowly deflated at approximately 2mmHg/sec until the Doppler signal reappeared. The pressure at which the Doppler signal reappeared was recorded as the brachial systolic pressure. Same procedure was repeated for the second arm and the highest brachial systolic pressure was used for ABI calculation [10].

In measuring the ankle pressure, the cuff was placed mid-way between the calf muscle and the malleolus. Ultrasound gel was applied to the posterior aspect of the medial malleolus. The transducer of the vascular Doppler was placed on the gel and its position was adjusted to the area with maximum signal intensity. The cuff of the sphygmomanometer was inflated to about 20mmHg above the expected systolic blood pressure of the patient. Then, the cuff was slowly deflated at approximately 2mmHg/sec until the Doppler signal reappears. The pressure at which the Doppler signal reappears was recorded as the ankle systolic pressure. Same procedure was repeated for the second ankle and the highest ankle systolic pressure was used for ABI calculation [10].


$$\text{ABI}=\frac{\text{Ankle Systolic Pressure}}{\text{Brachial Systolic Pressure}}$$

Calculated ABI value was recorded to two decimal places.

**Data analysis:** data analysis was done using Statistical Package for Social Sciences (SPSS) for Windows, version 20. Descriptive statistics was used to generate frequency table to determine the distribution of categories of foot-at-risk. The associations of foot-at-risk with the categorical variables were tested using either chi-square test or fisher's exact test. P-value was set at 0.05 (P-value = 0.05 was regarded as statistically significant).

### Operational definitions

***Glycaemic control:*** good glycaemic control implied glycosylated haemoglobin lesser than 7% while a value of = 7% was considered poor glycaemic control [11].

Loss of protective sensation (LOPS) was considered to be present when a patient has at least one abnormal test from the 10-g monofilament test and vibration test. The 10-g monofilament test was considered abnormal if total sensation from both feet is < 7/8. Vibration test was considered abnormal if there was absent sensation in at least one foot.

Definition of PAD was made by using ABI. It was considered to be present if ABI is either < 0.91 or > 1.30 [12]. Foot was considered to be deformed if there was presence of any of these; prominent metatarsals, callus, claw toes, hallux valgus, hammer toes and high arched feet. Participants were categorized into four categories as illustrated in Table 1.

**Table 1:** categories of foot-at-risk

Risk Category	Definition
0	No LOPS, No PAD, No deformity
1	LOPS ± deformity
2	PAD ± LOPS
3	History of ulcer or amputation

**Ethical consideration:** the study protocol was reviewed by the ethical committee of Bowen University Teaching Hospital, Ogbomoso and ethical approval was obtained before the study commenced. The study participants were required to sign the informed consent after the objectives and the procedure of the study were explained to them. All the consent forms and the questionnaires were kept confidential and used only for the study. This study protocol complied with the Declaration of Helsinki.

## Results

**Socio-demographic and medical characteristics of the participants:** the age group with the highest proportion of participants was age above 60 years with 41.8% (125) of the participants while the group below 30 years was the lowest with a frequency of 1.3% (4). Only 39.1% (117) of the participants were males and 60.9% (182) were females, giving a male to female ratio of 0.6: 1. Majority of the participants (274) were married. Christianity was the more practiced religion with a frequency of 76.3% (228). The largest proportion of the participants (285) belonged to Yoruba ethnic group. About 67.9% (203) of the respondents were in class 2 and majority of the participants (208) were urban dwellers. About 67.2% (201) of the participants had duration of diabetes less than 5 years. Among the participants, 51.5% (154) had co-existing medical illness. Of the co-existing medical conditions in the participants, hypertension ranked the highest with a prevalence of 43.5% (130). Other conditions reported included; osteoarthritis (5.7% (17)), asthma (1.0% (3)) and visual impairment (1.3% (4)) Table 2.

**Table 2:** socio-demographic and medical characteristics of the participants (N = 299)

	Frequency	Percentage (%)
**Age (years)**		
≤ 30	4	1.3
31-40	15	5.0
41-50	63	21.1
51-60	92	30.8
Above 60	125	41.8
**Mean age = 59.3**	Standard Dev. = 11.2	
**Gender**		
Female	182	60.9
Male	117	39.1
**Marital status**		
Single	3	1.0
Married	274	91.6
Separated	3	1.0
Widow	19	6.4
**Religion**		
Christianity	228	76.3
Islam	71	23.7
**Education**		
No formal	89	29.8
Primary	90	30.1
Secondary	55	18.4
Tertiary	65	21.7
**Social Class**		
Class 1	74	24.7
Class 2	203	67.9
Class 3	22	7.4
**Ethnicity**		
Yoruba	285	95.3
Hausa	3	1.0
Igbo	11	3.7
**Residence**		
Rural	91	30.4
Urban	208	69.6
**Cigarette Smoking**		
Yes	9	3.0
No	290	97.0
**Co-existing Condition**		
Absent	145	48.5
Hypertention	130	43.5
Osteoarthritis	17	5.7
Asthma	3	1.0
Visual Impairment	4	1.3
**Duration of Diabetes**		
≤ 5 years	201	67.2
6-10 years	48	16.1
Above 10 years	50	16.7

**Category of foot-at-risk in the participants:** majority of the participants (37.8% (113)) belonged to category 1 of foot-at-risk while the minimum number fell into category 3 with a frequency of 11.0% (33). Therefore, the prevalence of foot-at-risk in the study population was 64.9% (194) (Table 3).

**Table 3:** category of foot-at-risk in the participants

Categories of foot-at-risk	Frequency	Percentage (%)
0	105	35.1
1	113	37.8
2	48	16.1
3	33	11.0
Total	299	100.0

**Prevalence of the implicated risk factors:** the prevalence of foot deformity, peripheral neuropathy and peripheral arterial disease were 30.8% (92), 37.8% (113), and 16.1% (48) respectively. Callus accounted for the highest form of foot deformity in participants (16.4% (49)). Most of the participants had a poor glycaemic control with a frequency of 83.6% (250). The mean level of glycosylated haemoglobin (HbA1C) in this study was 9.2 ± 2.5%. The values of HbA1C of participants in this study ranged between 4.6 to 21% (Table 4).

**Table 4:** foot-at-risk factors in the participants (N = 299)

	Frequency	Percentage (%)
**LOPS**		
Absent	186	62.2
Present	113	37.8
**Deformity**		
Absent	207	69.2
Present	92	30.8
**Forms of Deformity**		
Nil	207	69.2
Callus	49	16.4
Hammer Toe	11	3.7
Claw Toes	6	2.0
Hallux Valgus	23	7.7
High Ached Feet	3	1.0
**PAD**		
Absent	251	83.9
Present	48	16.1
**Glycaemic Control**		
Poor Control	250	83.6
Good Control	49	16.4

**Factors associated with foot-at-risk:** Table 5 showed the associations of foot-at-risk with age group, religion, level of education, duration of care, smoking history and glycaemic control were found to be statistically significant.

**Table 5:** socio-medical factors associated with foot-at-risk in the participants (N = 299)

	0 N (%)	1 N (%)	2 N (%)	3 N (%)	Exact value	P-value
**Age Group (years)**						
≤ 30	3 (1.0)	1 (0.3)	0 (0.0)	0 (0.0)	21.273	0.047*
31-40	8 (2.7)	4 (1.4)	3 (1.0)	0 (0.0)		
41-50	26 (8.7)	24 (8.0)	6 (2.0)	7 (2.3)		
51-60	40 (13.4)	29 (9.7)	13 (4.4)	10 (3.3)		
Above 60	28 (9.3)	55 (18.4)	26 (8.7)	16 (5.4)		
**Religion**						
Christianity	80 (26.8)	87 (29.1)	28 (9.4)	33 (11.0)	22.087	0.001*
Islam	25 (8.3)	26 (8.7)	20 (6.7)	0 (0.0)		
**Level of Education**						
No formal	19 (6.3)	44 (14.8)	13 (4.4)	13 (4.4)	30.442	0.002*
Primary	26 (8.7)	36 (12.0)	15 (5.0)	13 (4.4)		
Secondary	22 (7.4)	18 (6.0)	12 (4.0)	3 (1.0)		
Tertiary	38 (12.7)	15 (5.0)	8 (2.7)	4 (1.2)		
**DM Duration**						
≤ 5	76 (25.4)	75 (25.1)	31(10.4)	17 (5.7)	25.916	0.001 *
Above 10	5 (1.7)	27 (9.0)	7 (2.3)	11 (3.7)		
Between 6-10	24 (8.0)	11 (3.7)	10 (3.4)	5 (1.6)		
**Glycaemic Control**						
Good control	12 (4.0)	22 (7.4)	15 (5.1)	0 (0.0)	18.040	0.001 *
Poor control	93 (31.1)	91 (30.4)	33 (11.0)	33(11.0)		
**Smoking History**					**12.907**	0.029*
Present	5 (1.7)	0 (0.0)	0 (0.0)	4 (1.3)		
Absent	100 (33.4)	113(37.8)	48 (16.1)	29 (9.7)		
**Total**	105 (35.1)	113 (37.8)	48 (16.1)	33 (11.0)		

(*) - Statistically Significant Bold values – Fisher?s exact test

## Discussion

The age group above 60 years had the highest proportion of respondents, which was 41.8% while the age group below or equal to 30 years was the lowest, which was 1.3%. The minimum age in the participants was 30 years while the maximum was 85 years and the mean age was 59.3 ± 11.2 years. The age distribution of the respondents in this study illustrated that majority of the participants with DM are usually in the older age group since the risk of Type 2 DM increases which is the commonest increases with age. The age distribution finding in this study aligned with other related studies [6,13-16].

Majority of the respondents were females with a prevalence of 60.9% and a male-to-female ratio of 0.6: 1. The larger proportion of female in the study reflected the positive attitude of females to seeking health care. However, the report by Ogbera *et al*. [6] revealed that males were more than females (1: 0.97). This may be attributed to unhealthy lifestyle lived by urban male dwellers because the study was conducted in Lagos. In this study, 76.3% of the respondents were Christians. Ogbomoso is generally known to be a Christian community that might have accounted for the above finding. However, previous similar studies did not emphasize this and this may be important in foot-at-risk and foot care practice. It can be seen that 51.5% of the respondents had co-existing medical conditions. The identified medical conditions included; hypertension, asthma, visual impairment and osteoarthritis. However, the condition with the highest proportion was hypertension which was seen in 43.5% of the participants. In this study, 67.2% of the respondents had duration of DM treatment less than 5 years while a smaller percentage (16.7%) had duration of treatment over 10 years. The shorter duration of treatment seen in the respondents may be due to late presentation to the hospital for diagnosis and treatment. The late presentation may also be attributed to the cultural perception of patients and the low level of education reported earlier. However, Ogbera *et al*. [6] reported that 40.6% of the respondents had medium term DM duration (5-9 years). This may be due to higher level of education seen in urban dwellers. More than half of the participants had foot-at-risk with a prevalence of 64.9%. This prevalence was distributed across the different categories with the highest in category 1. The distribution included; 35.1% in category 0, 37.8% in category 1, 16.1% in category 2, and 11% in category 3. In this study, a high prevalence of foot-at-risk may be due to the increase in the prevalence of diabetes with resultant increase in its complications. Conversely, Ogbera *et al*. [6] in a study done in Lagos to assess the level of foot-at-risk, reported the prevalence of foot-at-risk as 41.5%. However, the foot-at-risk was only identified but not categorized. This may be because the study had earlier been done before the comprehensive foot examination and categorization was released in 2008. Other similar studies reported a prevalence of 41.4% with category 3 being the highest (17.3%) [15]; 52% majority of the participants belonged to category 0 (48%) [17]; 15.3% with majority in category 1 [13].

As earlier highlighted, the common risk triad in the development of foot ulceration in DM patients is peripheral neuropathy, peripheral arterial disease and foot deformity [18]. Peripheral neuropathy was seen in 37.8% of the participants. The level of peripheral neuropathy seen in the study population may be because the majority of the participants belonged to age range over 60 years. A similar prevalence was reported by Ogbera *et al*. [12]. In addition, the level of peripheral neuropathy reported in this study and others may be due to poor glycaemic control as well as coexisting conditions like hypertension. Peripheral arterial disease was assessed with a vascular Doppler in this study. The prevalence of PAD in this study was 16.1%. However, Ogbera *et al*. [12] reported PAD prevalence as 40%. A vascular Doppler with frequency of 12mHz was used to assess PAD contrary to the conventional 8mHz used for this study. So, this may be responsible for higher prevalence of PAD reported. Kishore *et al*. [17] reported a prevalence of 10% for PAD. The varying levels of PAD in the reported studies may be related to the poor metabolic control of the participants and other factors like, coexisting conditions, assessment tools, assessors' expertise and so on. The prevalence of foot deformity in this study was 30.8%. Among the study participants, callus accounted for 16.4%, hammer toe accounted for 3.7%, claw toes accounted for 2.0%, hallux valgus was seen in 7.7% and 1.0% had high-arched feet. The higher prevalence of callus seen may be due to the advanced age of the participants, the geographical location of the study area and the social class of the participants.

There was an unacceptable poor glycaemic control in participants of as low as 16.4% good control level. The mean glycaemic control was 9.2%. A larger portion of participants with good glycaemic control were located in category 1 of foot-at-risk with a percentage of 44.9%. In addition, the group with worst glycaemic control was category 3 with none of the participants with good control. In other words, there was a trend between the increasing severity of the category of foot-at-risk and glycaemic control. The association between foot-at-risk and glycaemic control was statistically significant (P-value = 0.001). As earlier noted, the prevalence of foot-at-risk in this study was 64.9%, the poor glycaemic control among participants might have accounted for the high prevalence of foot-at-risk. Consistent with the present study, several studies have reported similar outcome [6,12,19]. The proportion of foot-at-risk in all categories increased with age. Majority of those with foot-at-risk belonged to the age group above 60 years. The association of foot-at-risk with age was statistically significant (P-value = 0.047). In other words, increase in age has the tendency to increase the risk of foot ulcer. The above finding may be due to the risk of development of both neuropathy and arterial disease with advancing age. This assumption was supported by Ogbera *et al*. [12] that reported that older age of > 60 years and poor glycaemic control were potential predictors of neuropathy.

Majority of the study participants (67.2%) had duration of diabetes mellitus less than 5 years. Among the participants with diabetes less than 5 years, 61.8% of them had foot-at-risk. The association of foot-at-risk with duration of diabetes was found to be statistically significant (P-value = 0.001). Similarly, Shahbazian *et al*. [20] in Iran reported that there was statistically significant association between higher foot-at-risk and longer diabetes duration (P-values = 0.001). Alam *et al*. [21] reported that longer duration of diabetes greater than 10 years was a significant risk factor in the development of diabetic foot complications. Other factors that were found to have statistically significant association with foot-at-risk included; religion (P-value = 0.001), level of education (P-value = 0.002), and history of smoking (P-value = 0.029). Few Muslims had foot-at-risk from this study. This may be associated with the good foot care activities practiced by the religion. The level of education of the patients may affect both the understanding of foot care education, foot care practice, and their glycaemic control activities.

**Study limitation:** since this study was hospital-based and not multicenter study, with a low response from other ethnic groups in the country the result may not be representative of what obtains in the general population.

## Conclusion

This study confirmed the alarming burden of foot-at-risk in patients with diabetes mellitus attending the outpatient clinics in Bowen University Teaching Hospital, Ogbomoso. Majority of the patients in this study population are at considerable risk of developing foot ulcers. In view of this, foot screening should be done by all clinicians routinely for DM patients. In addition, patients identified should be managed and followed up appropriately [9]. One of the important factors in the genesis of DFD is poor glycaemic control. Unfortunately, the long-term glycaemic control of the participants was unacceptably poor. This finding showed that there is need to intensify efforts targeting good glycaemic control in the patients with DM in order to prevent complications from the disease. The socio-medical factors associated with foot-at-risk in this study were age, religion, level of education, duration of diabetes, and history of smoking.

### What is known about this topic

- *Foot-at-risk has been known to be the predecessor of diabetic foot disease, which may progress to foot amputation; as shown in different classification of diabetic foot;*
- *The burden of foot-at-risk is high in South-west, Nigeria as reported by Ogbera et al*[6].

### What this study adds

- *Even though foot-at-risk is known to precede diabetic foot, researchers have only concentrated on DM foot studies; in addition, there is paucity of data on foot-at-risk in South-west, Nigeria; the few available studies are old and standardized tools were not used for assessment of foot-at-risk;*
- *This study reviewed the current profile of foot-at-risk in DM patients in the South-western, Nigeria using a standardized tool;*
- *This study emphasized on the need for proactive actions by both clinician and DM patients towards prevention of DM foot*.
